# Influence of pupil dilation on the Barrett universal II (new generation), Haigis (4th generation), and SRK/T (3rd generation) intraocular lens calculation formulas: a retrospective study

**DOI:** 10.1186/s12886-020-01571-1

**Published:** 2020-07-20

**Authors:** Takeshi Teshigawara, Akira Meguro, Nobuhisa Mizuki

**Affiliations:** 1 Department of Ophthalmology, Yokosuka Chuoh Eye Clinic, 238-0008, 2-6 Odaki-cho, Yokosuka, Kanagawa Japan; 2 Department of Ophthalmology, Yokohama Tsurumi Chuoh Eye Clinic, Yokohama, Kanagawa Japan; 3 Department of Ophthalmology, Yokohama City University School of Medicine, Yokohama, Kanagawa Japan

**Keywords:** Anterior chamber depth, Barrett, Haigis, Intraocular lens, Lens thickness, Predicted postoperative refraction, Pupil dilation, SRK/T, White-to-white

## Abstract

**Background:**

Despite the surge in the number of cataract surgeries, there is limited information available regarding the influence of pupil dilation on predicted postoperative refraction and its comparison with recommended various intraocular lens power calculated using the different parameters. We used three different IOL power calculation formulas: Barrett Universal II (Barrett) (5-variable formula), Haigis (3-variable formula), and SRK/T (2-variable formula), in order to investigate the potential effect of pupil dilation on the predicted postoperative refraction (PPR) and recommended intraocular lens (IOL) power calculation.

**Methods:**

This retrospective study included 150 eyes. All variables were measured and calculated using a ZEISS IOL Master 700. The following variables were measured before and after dilation: anterior chamber depth (ACD), lens thickness (LT), white-to-white (WTW). PPR and recommended IOL power were calculated by Barrett, Haigis, and SRK/T IOL calculation formulas. The change in each variable before and after dilation, and the correlations between all changes were analyzed using the Wilcoxon signed-rank test and the Spearman’s rank-order correlation test, respectively.

**Results:**

The mean absolute change (MAC) in PPR before and after dilation was found to be highest in the Barrett formula. Significant differences were found between each MAC (*P* <  0.0001). Significant changes were observed before and after dilation in ACD and LT (*P* <  0.0001), but not in WTW. Using the Barrett and Haigis formulas, there was a significant positive correlation between the change in PPR and change in ACD (*P* <  0.0001), and a negative correlation between change in PPR and change in LT (*P* <  0.0001). The correlations were strongest with the Barret formula followed by the Haigis, particularly in terms of LT. Changes in PPR determined by the Barrett formula also demonstrated a significant positive correlation with changes in WTW (*P* = 0.022). The recommended IOL power determined using Barrett and Haigis changed before and after dilation in 23.3 and 19.3% cases respectively, while SRK/T showed no change.

**Conclusions:**

In terms of PPR and recommended IOL power, pupil dilation influenced mostly the Barrett formula. Given the stronger correlation between the changes in PPR when using Barrett and the changes in ACD, LT, and WTW, changes in ACD, LT, and WTW significantly affect how dilation influences the Barrett formula. Determining how dilation influences each formula and other variables is key to improving the accuracy of IOL calculations.

## Background

As patient expectations on the outcome of cataract surgery increase, ophthalmologists need to pay special attention to the accuracy of predicted postoperative refraction (PPR). Various intraocular lens (IOL) calculation formulas are available, such as the 2-variable formula SRK/T [1]; the 3-variable formula Haigis [2]; and the 5-variable formula Barrett Universal II [3]. Many research papers studying the accuracy of the predictability of different IOL calculation formulas have been published [4, 5]. Most researchers state that Barrett Universal II is one of the most reliable IOL calculation formulas [4, 5]. Several studies have analyzed the influence of preoperative anterior chamber depth (ACD) on PPR in different IOL calculation formulas, and concluded that the influence of pre-operative ACD on PPR, varies from formula to formula [6, 7]. Further studies have examined the influence of pupil dilation on biometric parameters such as ACD, lens thickness (LT), white-to-white (WTW), and recommended IOL power using different IOL calculation formulas [8–10]. Thus, there is only a small number of research papers that have compared the influence of pupil dilation on PPR and recommended IOL power calculated using the different types of IOL calculation formulas, and different biometric parameters, such as ACD, LT and WTW. However, to the best of our knowledge, this is the first study that has investigated the correlation between PPR and recommended IOL power in three different IOL calculation formulas, as well as the changes in the biometric parameters, ACD, LT and WTW. Given that different IOL calculation formulas include different biometric parameters, and can be influenced by pupil dilation, further research in this area is necessary.

The purpose of this study was to analyze the influence of pupil dilation on biometric variables and recommended IOL power calculated using the Barrett Universal II, Haigis, and SRK/T formulas. Additionally, the correlation between all variables was investigated.

## Methods

This was a retrospective study which included 150 eyes of 81 patients. Cataract operations without any unexpected events were performed at two eye clinics (Yokosuka Chuoh Eye Clinic and Tsurumi Chuoh Eye Clinic). For all patients, monofocal acrylic single piece IOLs (SN60WF, Alcon Laboratories, Inc., Fort Worth, TX, USA) were inserted.

The study adhered to the tenets of the Declaration of Helsinki throughout the entire data collection process, and ethical committees of both eye clinics approved the study. Consent to use their medical data for this research was given by all participating patients whose postoperative best-corrected visual acuity was better than 20/40 without any history of eye problems and intraocular, or corneal operations.

All biometric variables, including ACD, LT, WTW, PPR, and recommended IOL power, were measured and calculated before and after pupil dilation using a ZEISS IOL Master 700 (Carl Zeiss Meditec AG, Jena, Germany). PPR and recommended IOL power were calculated using three different IOL power calculation formulas: Barrett Universal II (5-variable formula), Haigis (3-variable formula), and SRK/T (2-variable formula) for SN60WF (Alcon Laboratories, Inc.), using a constant of 119.0 provided by the User Group for Laser Interference Biometry (ULIB). Lens constant optimizations for SN60WF were performed in collaboration with IOL Master 700 which has licensed versions of the proprietary Barrett Universal II. However, the lens constants we used for Haigis and SRK/T were the already optimized values for IOL Master 700 as listed on the ULIB website. The lens factor for Barrett Universal II was 1.94, the a0, a1, and a2 constants for Haigis were − 1.268, 0.342, and 0.233, and the A-constant for SRK/T was 119.1.

After the pre-dilation examination, topical tropicamide and phenylephrine (Midrin-P®, Santen, Osaka, Japan) were applied every 15 min. After full dilation, which was defined as a diameter of at least 6 mm, the post-dilation examination was performed.

The mean change in ACD, LT, and WTW, and the mean absolute change (MAC) in PPR for each formula were analyzed. The correlation between the aforementioned variables, was also investigated. Additionally, the difference between the coincidence rate of recommended IOL power for each formula before and after pupil dilation was assessed. Finally, based on the collected data, the influence of pupil dilation on all variables was analyzed.

The Wilcoxon signed-rank test was used to compare changes in ACD, LT, and WTW and change in PPR for each formula before and after dilation. Spearman’s rank-order correlation test was used to investigate the correlation of these variables. A difference in recommended IOL power within ±0.5D was regarded as coinciding. Fisher’s exact test was used to compare the recommended IOL power. A value of *P* < 0.05 was considered as statistically significant. The Bell Curve for Excel, version 1.03 (Social Survey Research Information Co, Ltd., Tokyo, Japan) was used to analyze statistical data.

## Results

A total of 150 eyes of 81 patients were included in this study. Mean patient age was 72.9 ± 7.7 years old (Table 1). In addition, the mean pre-dilation ACD, LT, and WTW values were 3.08 ± 0.40 mm (range: 2.08–4.28 mm), 4.57 ± 0.46 mm (range: 3.44–5.87 mm), and 11.87 ± 0.37 mm (range: 10.8–12.8 mm), respectively.

**Table 1:** Clinical characteristics of our study population

	Mean	SD	Min	Max
Age	72.9	7.7	51	87
Males N (%)	32 (39.6)	NA	NA	NA
ACD	3.08	0.4	2.01	4.28
LT	4.57	0.46	3.44	5.87
WTW	11.87	0.37	10.8	12.8

Table 2 indicates the influence of pupil dilation on ACD, LT, and WTW. ACD and LT significantly changed after dilation (*P* < 0.0001), but WTW did not.

**Table 2:** Effect of pupil dilation on anterior chamber depth, lens thickness, and white-to-white

Parameters	Mean^a^, mm		Mean difference post- minus pre-dilation, mm	Number of eyes			
	Pre-dilation	Post-dilation		D^b^ < 0	D = 0	D > 0	*P*
ACD	3.08 ± 0.40	3.14 ± 0.41	0.06 ± 0.03	0 (0.0%)	0 (0.0%)	150 (100.0%)	< 0.0001
LT	4.57 ± 0.46	4.55 ± 0.41	−0.02 ± 0.01	124 (82.7%)	24 (16.0%)	2 (1.3%)	< 0.0001
WTW	11.87 ± 0.37	11.88 ± 0.38	0.02 ± 0.11	49 (32.7%)	39 (26.0%)	62 (41.3%)	0.16

^a^Data are presented as means ± standard deviations

^b^D is the difference post- minus pre-dilation

*ACD* Anterior chamber depth, *LT* Lens thickness, *WTW* White-to-white

**Fig. 1:**
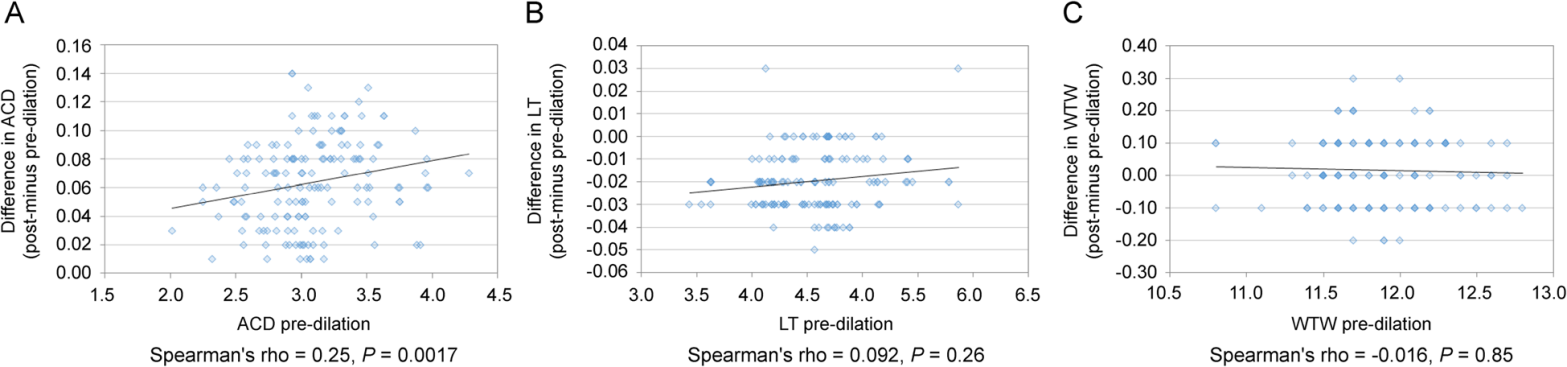
Correlation between change in ACD (**a**), LT (**b**), and WTW (**c**) and pre-dilation ACD (**a**), LT (**b**), and WTW (**c**)

There was a significant positive correlation between pre-dilation ACD and change in ACD (Spearman’s rho = 0.25, *P* = 0.0017); however, there was no significant correlation between pre-pupil dilation and change in LT, while the same was observed forWTW [Spearman’s rho = 0.092, *P* = 0.26, and Spearman’s rho = − 0.016, *P* = 0.85, respectively (Fig. 1)]. Mean absolute change in PPR calculated with each formula is shown in Table 3. The mean absolute change in PPR using the Barrett Universal II was highest (0.047 ± 0.029), followed by the Haigis (0.035 ± 0.019), and SRK/T (0.0052 ± 0.0053) formulas. Significant differences were found among each MAC in terms of PPR (*P* < 0.0001).

**Table 3:** Mean absolute change in predicted postoperative refraction between pre- and post-pupil dilation calculated by the three formulas

		***P value***		
Formulae	Mean absolute change^a^	SRK vs Haigis	Haigis vs Barret	SRK vs Barret
SRK/T	0.0052 ± 0.0053 D [0.00–0.11]	< 0.0001	< 0.0001	< 0.0001
Haigis	0.035 ± 0.019 D [0.00–0.08]			
Barrett	0.047 ± 0.029 D [0.00–0.02]			

^a^Data are presented as means ± standard deviations

**Fig. 2:**
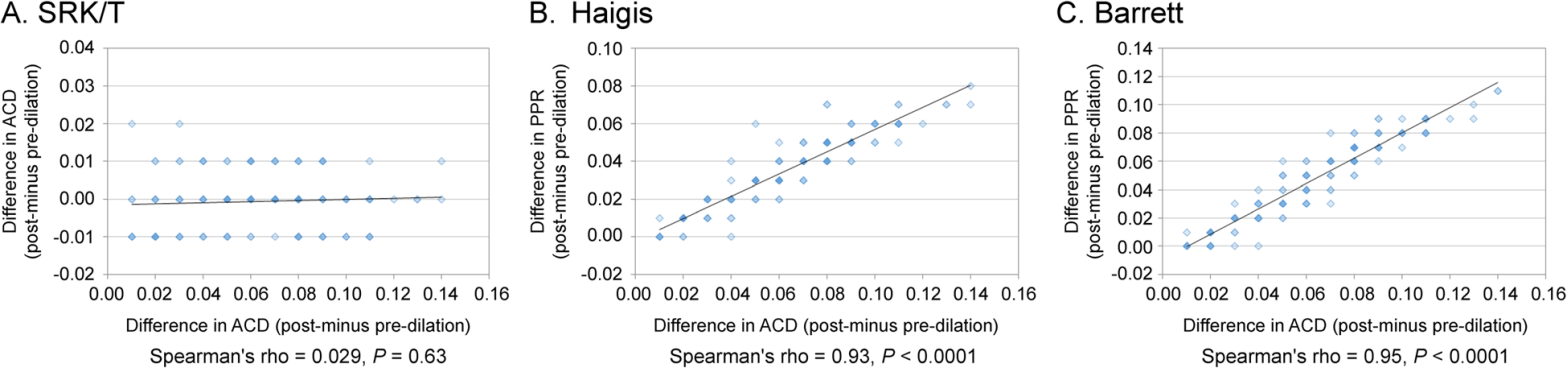
Correlation between change in ACD and change in PPD in SRK/T (**a**), Haigis (**b**), and Barrett (**c**)

**Fig. 3:**
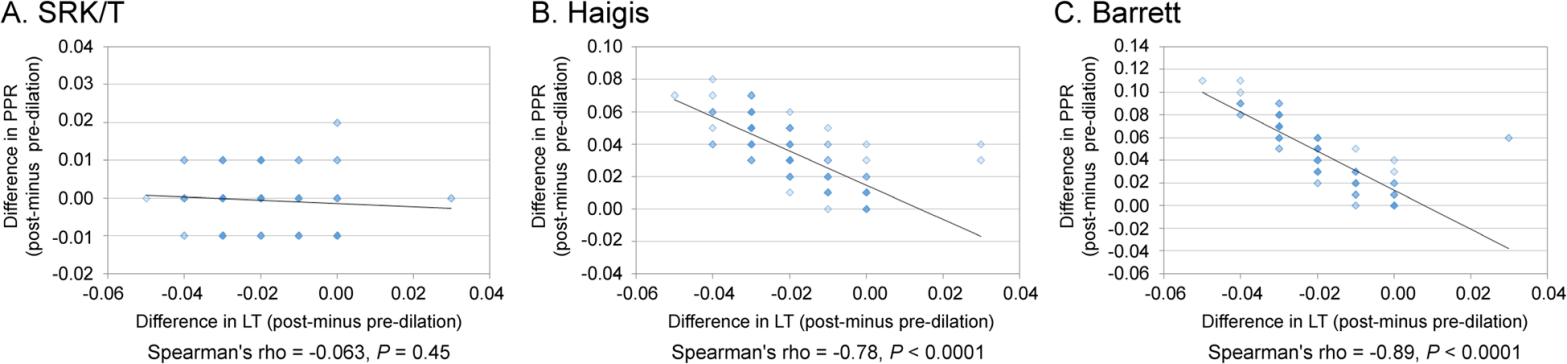
Correlation between change in LT and change in PPD in SRK/T (**a**), Haigis (**b**), and Barrett (**c**)

**Fig. 4:**
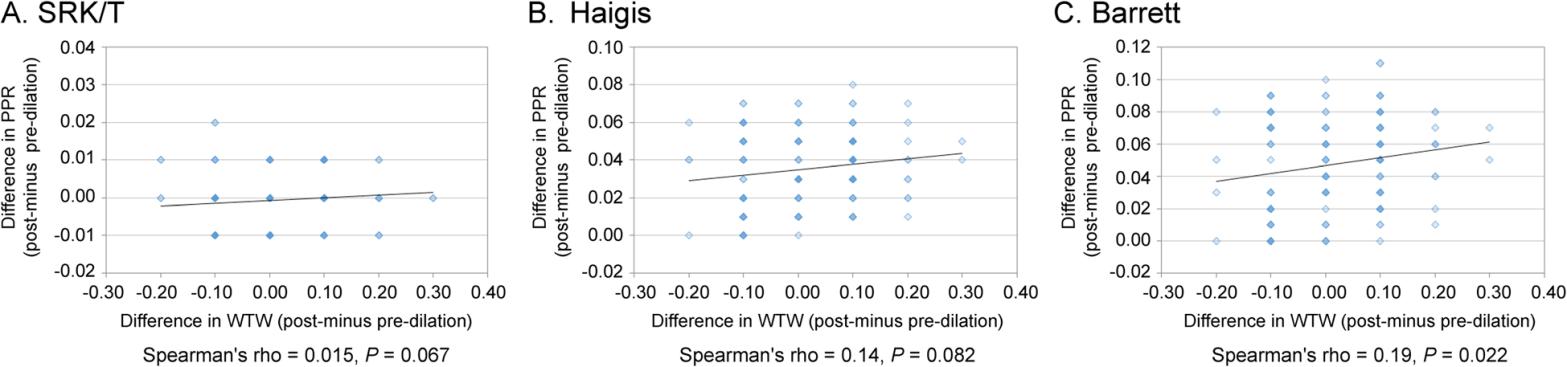
Correlation between change in WTW and change in PPD in SRK/T (**a**), Haigis (**b**), and Barrett (**c**)

**Fig. 5:**
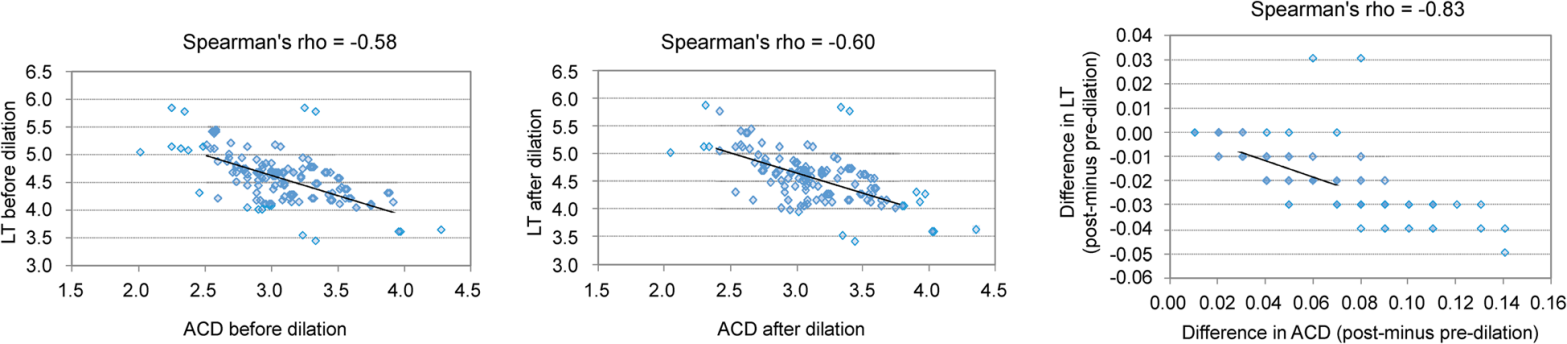
Correlation between ACD and LT pre- and post-dilation and their difference (post- minus pre-dilation)

Using the Barrett Universal II and Haigis formulas, there was a significant positive correlation between the change in PPR and change in ACD (Spearman’s rho = 0.95, *P* < 0.0001, and Spearman’s rho = 0.93, *P* < 0.0001 respectively); however, this correlation was not observed with SRK/T (Spearman’s rho = 0.029, *P* = 0.63) (Fig. 2). On the other hand, when using Barrett Universal II and Haigis, there was a significant negative correlation between change in PPR and change in LT (Spearman’s rho = − 0.89, *P* < 0.0001, and Spearman’s rho = − 0.78, *P* < 0.0001, respectively); but this tendency was not observed with SRK/T (Spearman’s rho = − 0.063, *P* = 0.45) (Fig. 3). There was a significant positive correlation between the change in PPR and change in WTW when using the Barrett Universal II (Spearman’s rho = 0.19, *P* = 0.022); this correlation was not found with Haigis and SRK/T (Spearman’s rho = 0.14, *P* = 0.082, and Spearman’s rho = 0.15, *P* = 0.067, respectively) (Fig. 4). Finally, we assessed whether the changes in LT and ACD were correlated. ACD and LT showed significant negative correlations with both pre- and post-pupil dilation (Spearman’s rho = − 0.58 and − 0.60 respectively) (*P* < 0.0001). As ACD deepened after pupil dilation, LT became significantly thinner (Spearman’s rho = − 0.83) [*P* < 0.0001, (Fig. 5)].

The coincidence rates of recommended IOL power before and after pupil dilation in each formula are displayed in Table 4. The recommended IOL power changed after dilation in 23.3% of cases when using Barrett Universal II and in 19.3% of cases using Haigis. In all cases, recommended IOL power coincided before and after dilation when using SRK/T. The inconsistency rate when using Barrett Universal II and Haigis was significantly higher than SRK/T (*P* < 0.0001). The recommended IOL power changed more frequently with Barrett Universal II than with Haigis; however, the difference in coincidence rate was not significant.

**Table 4:** Coincidence of recommended IOL power between pre- and post-pupil dilation in the three formulas

	Number of eyes		
	SRK/T	Haigis	Barrett
Coincidence	150 (100.0%)	121 (80.7%)	115 (76.7%)
	0 (0.0%)	29 (19.3%)	35 (23.3%)

*P* < 0.0001 for SRK/T vs. Haigis or Barrett, and *P* = 0.48 for Haigis vs. Barrett

*IOL* Intraocular lens; SRK/T

## Discussion

In this study, PPR and the recommended IOL power were differently influenced by pupil dilation when calculated using three different formulas. Of those tested in this study, Barrett Universal II, a 5-variable formula, was the most sensitive to pupil dilation, followed by Haigis, whereas SRK/T was not influenced by pupil dilation. The change in ACD and LT before and after pupil dilation were mostly influenced by Barrett Universal II and Haigis. The change in WTW before and after pupil dilation only influenced Barrett Universal II. Improvements in the accuracy of biomechanical measurements and PPR have gained attention, as they are important in choosing the most suitable IOL [4, 5]. Therefore, we must consider all factors that influence these measurements as well as PPR.

Different IOL calculation formulas, include different biomechanical parameters to estimate the effective lens position (ELP), an important factor for PPR [1–3]. SRK/T uses corneal curvature radius and axial length to estimate ELP, which was determined by Retzlaff et al. in 1990 [1]. Effective lens position is estimated based on ACD and AL in Haigis [2], and Barrett Universal II uses AL, corneal curvature radius, ACD, LT, and WTW [3]. Several studies have investigated the influence of pupil dilation on these biometric measurements [8, 10–16]. In a clinical setting, pupil dilation is a vital process of preoperative examination. Therefore, it is important to analyze its possible influence on PPR and recommended IOL power in different power calculation formulas, and to investigate the correlation between variables.

Previous studies, reported that AL and corneal curvature radius are not affected by pupil dilation [10–12] as has been the case for pupil dilation [10–14]. Compared to ACD, few studies have dealt with the influence of pupil dilation on LT and WTW. Wang X et al. [8] demonstrated that LT was significantly affected from it. The influence of pupil dilation on ACD and LT is sensible because the ciliary and dilator muscles relax and contract, respectively, causing the lens to become thinner and the ACD to become deeper. This phenomenon was observed in this study as well and it occurs because as the ciliary muscles relax due to pupil dilation, the tension on the zonules increases, the lens becomes thinner, and as a result, ACD deepens [17]. Controversy exists on whether pupil dilation influences WTW. While Huang et al. [10] and Arriola-Villalobos et al. [15] have insisted that WTW is affected by pupil dilation, the opposite result has been reported by Wang et al. [8] Although the researchers attributed the discrepancy in the influence of pupil dilation on WTW to the examination error and imaging artifact [8], the real mechanism remains unknown.

In our study, while ACD significantly increased after dilation, LT significantly decreased, which are both consistent with previous research [16]. WTW did not significantly change. Regarding the influence of pupil dilation on PPR and recommended IOL power, the outcomes of previous studies are inconsistent, and vary among formulas [10, 12, 16, 18, 19].

Rodriguez-Raton et al. showed that PPR was not affected by pupil dilation when using SRK/T, but was, when using Haigis [12]. Adler et al. indicated similar results [18]. These results were reasonable since SRK/T does not include ACD as a biometric parameter, which is significantly affected by pupil dilation, whereas, Haigis does. Our research also showed that while PPR did not change after pupil dilation when using SRK/T, but did when using Haigis. Regarding Barrett Universal II, although many studies have demonstrated its superior accuracy in calculating PPR compared to other formulas [4, 5], studies investigating the influence of pupil dilation on PPR and recommended IOL power on Barrett Universal II, have not been published. Our research indicated that the mean change in PPR was largest when calculated using Barrett Universal II, followed by Haigis, and SRK/T. This suggests that Barrett Universal II was the most sensitive to pupil dilation. A positive change indicates that the formula predicts a more hyperopic result for a given IOL power. Therefore, as ACD increased, the Barrett and Haigis formulae predicted a more hyperopic postoperative refraction. The difference in the sensitivity to pupil dilation among formulas was significant. The recommended IOL power calculated with Barrett Universal II changed mostly among formulas, although it was not statistically significant between Barrett Universal II and Haigis. Although several studies have demonstrated that the recommended IOL power calculated with Haigis, is significantly affected by pupil dilation, but not if calculated using SRK/T [10, 12, 16], our research is the first to show that Barrett Universal II may be even more sensitive to pupil dilation than Haigis, considering PPR and recommended IOL power.

The analysis of the correlation between the change in PPR and the biometric variables indicated that the 5-variable formula is more sensitive to pupil dilation. The change in PPR determined using Barrett Universal II and Haigis showed a positive correlation with the change in ACD and a negative correlation with the change in LT, but this was not with SRK/T. This result indicated that the change in ACD and LT significantly influenced the change in PPR in the formulas, which included ACD as a biometric parameter. Additionally, the change in PPR when using Barrett Universal II indicated a significant positive correlation with the change in WTW, but not when using Haigis and SRK/T. This outcome was persuasive since Barrett Universal II was the only formula that included WTW as a biometric factor. Given the fact that all biometric factors could be significantly influenced by pupil dilation, it is convincing that the more biometric parameters an IOL calculation formula includes, the more influential pupil dilation is on the formula. As a result, recommended IOL power calculated by Barrett Universal II, which is a 5-variable formula, changed in more cases after pupil dilation compared to Haigis and SRK/T.

Thus, there are biometric factors in the IOL calculation formula that are influenced by pupil dilation. Barrett Universal II is said to be one of the most reliable IOL calculation formulas [4, 5]. However, this study demonstrated that since it includes more biometric variables compared to formulas with fewer, eye specialists must be familiar with these phenomena to improve the accuracy of IOL calculation.

The results of this study, are not only statistically, but also clinically significant as well. A statistically significant change in PPR determined by Barrett Universal II and Haigis between pre- and post-pupil dilation can influence a physicians’ choice of the IOL power. Given the fact that the post-operative refractive error is the most common cause of patient dissatisfaction especially in the case of multifocal IOL, it is crucial to take this information into consideration [20].

Despite the advantages mentioned above, this study has some limitations. First, the results of our study can’t be generalized due to the ocular characteristics of Asian populations [21, 22]. Furthermore, the inclusion of data from both eyes of some patients in the study may have had a coupling effect in the statistical analysis. Third, different surgeons performed the surgeries, which may have affected the postoperative IOL position. Finally, the influence of pupil dilation on prediction error in refraction was not analyzed, which would enable optimization of the constant for measurement with or without pupil dilation. This idea would be useful to improve the accuracy of IOL power calculations. We plan to analyze this in future research.

## Conclusions

In our study, pupil dilation influenced mostly Barrett, followed by Haigis and SRK/T, in terms of both PPR and recommended IOL power. Given the stronger correlation between the change in PPR when using Barrett and the change in ACD, LT, and WTW, the change of ACD, LT, and WTW significantly affect how dilation influences the Barrett formula. The influence of dilation on each formula and variables including ACD, LT, and WTW is key to improving the accuracy of IOL calculations.

## Data Availability

The datasets used and/or analysed during the current study are available from the corresponding author upon reasonable request.

## Abbreviations

ACD: Anterior chamber depth
AL: Axial length
ELP: Effective lens position
IOL: Intraocular lens
LT: Lens thickness
MAC: Mean absolute change
PPR: Predicted postoperative refraction
WTW: White-to-white
